# Innovative high-resolution microCT imaging of animal brain vasculature

**DOI:** 10.1007/s00429-020-02158-8

**Published:** 2020-10-31

**Authors:** Ruslan Hlushchuk, David Haberthür, Petr Soukup, Sebastien F. Barré, Oleksiy-Zakhar Khoma, Johannes Schittny, Neda Haghayegh Jahromi, Audrey Bouchet, Britta Engelhardt, Valentin Djonov

**Affiliations:** 1 Institute of Anatomy, University of Bern, Bern, Switzerland; 2 Theodor-Kocher Institute, University of Bern, Bern, Switzerland; 3 Ophthalmology Section, Equine Department, Vetsuisse Faculty, University of Zurich, Zurich, Switzerland; 4 Roche Pharmaceutical Research and Early Development, Pharmaceutical Sciences, Roche Innovation Center, Basel, Switzerland; 5 Synchrotron Radiation for Biomedicine Group (STROBE), Université Grenoble Alpes, UA7, Grenoble, France

**Keywords:** microCT, microangioCT, Visualization of the vasculature, Blood vessels, Angiogenesis

## Abstract

Analysis of the angioarchitecture and quantification of the conduit vessels and microvasculature is of paramount importance for understanding the physiological and pathological processes within the central nervous system (CNS). Most of the available in vivo imaging methods lack penetration depth and/or resolution. Some ex vivo methods may provide better resolution, but are mainly destructive, as they are designed for imaging the CNS tissues after their removal from the skull or vertebral column. The removal procedure inevitably alters the in situ relations of the investigated structures and damages the dura mater and leptomeninges. µAngiofil, a polymer-based contrast agent, permits a qualitatively novel postmortem microangio-computed tomography (microangioCT) approach with excellent resolution and, therefore, visualization of the smallest brain capillaries. The datasets obtained empower a rather straightforward quantitative analysis of the vascular tree, including the microvasculature. The µAngiofil has an excellent filling capacity as well as a radio-opacity higher than the one of bone tissue, which allows imaging the cerebral microvasculature even within the intact skull or vertebral column. This permits in situ visualization and thus investigation of the dura mater and leptomeningeal layers as well as their blood supply in their original geometry. Moreover, the methodology introduced here permits correlative approaches, i.e., microangioCT followed by classical histology, immunohistochemistry and even electron microscopy. The experimental approach presented here makes use of common desktop microCT scanners, rendering it a promising everyday tool for the evaluation of the (micro)vasculature of the central nervous system in preclinical and basic research.

**Electronic supplementary material:**

The online version of this article (10.1007/s00429-020-02158-8) contains supplementary material, which is available to authorized users.

## Introduction

The central nervous system is essential for higher organisms and proper blood supply is critical for its functioning. Understanding the angioarchitecture of the conduit vessels and of the microvascular bed is of paramount importance to understand the metabolic activity and function of the nervous system (Quintana et al. 2019). Many physiological and pathological processes in the central nervous system involve changes in the vasculature, starting from the macroscopic level (e.g., aberrant vessels, subarachnoid hemorrhage, arteriovenous malformations, and cavernous malformations) down to the microvessels and capillaries (e.g., cerebral small-vessel disease) (Frosen and Joutel 2018; Heinzer et al. 2006). Alterations of the vascular structure/morphology are responsible for the etiology of clinically relevant brain pathologies, such as intracranial tumors, stroke, dementia, Alzheimer’s disease, microbleeds, and cortical atrophy (Blinder et al. 2010; Hirsch et al. 2012; Meyer et al. 2008; Ostergaard et al. 2016; Pantoni 2010; Xie et al. 2012; Xiong et al. 2017). The number of patients that would potentially benefit from a better understanding of the vasculature alterations during pathophysiological processes is extremely high. Therefore, a lot of effort has been put into the investigations of the vasculature of the central nervous system in preclinical animal models. In the last decades, a few imaging modalities arose with a combination of great advantages and drawbacks.

Most of the in vivo imaging methods simply lack the resolution or penetration depth rendering them hardly usable for the analysis of the microvasculature throughout the brain. Probably, the most powerful in vivo method with good penetration is a contrast-enhanced in vivo microCT. In vivo microCT scanners are able to reliably visualize medium to large vessels, but they cannot be used to analyze microvessels, which are below the resolution of the imaging system (around 50 µm) (Demene et al. 2016). The analysis of small vessels is essential for vascular pathologies, as changes in the microvasculature often reflect on all the hierarchical structure of blood vessels (Nebuloni et al. 2014).

Most of the current ex vivo methods are able to provide the resolution needed for the visualization of capillaries. A serious disadvantage of most such imaging techniques is their organ destructive nature (e.g., serial sectioning or vascular corrosion casting, followed by scanning electron microscopy or microCT) or the need for expensive and rare equipment or experimental time (e.g., synchrotron radiation-based microtomography).

The development of the treatment strategies targeting pathological processes associated with vasculature needs a robust imaging approach enabling a preferably non-destructive, 3D-imaging of the macro- and microvasculature with the highest possible resolution. The methodology should provide datasets that can be analyzed both qualitatively and quantitatively. Additionally, it should be possible to correlate these three-dimensional data sets with the local morphology, i.e., enable a correlative imaging approach.

To reach the aforementioned higher resolution, a postmortem microangiography has often been performed in combination with vascular corrosion casting. In this approach, the vasculature is filled with a casting agent that solidifies within the vascular lumen (any discontinuity of the filling results in “loss” of vascular segments and, therefore, compromises all-inclusive visualization and reliable quantification). The surrounding tissue is afterward macerated away with an alkaline solution. The remaining cast represents the filled lumen of the original vasculature and can then be imaged in a microCT. Due to the fact that the resulting sample to be scanned is essentially only the corrosion cast and air, good signal-to-noise ratio and thus high-quality images can be gained from this method (Meyer et al. 2008; Quintana et al. 2019). The aforementioned corrosion vascular casting method, even if combined with microCT, is nonetheless a destructive method, rendering any further histological or ultrastructural investigation impossible. Additionally, even with this approach, the visualization of the vessels within the bone remains a challenge (Schneider et al. 2009).

By employing contrast agents such as barium sulfate suspension in gel or Microfil, it is both possible to overcome the destructiveness of the approach and to enhance the signal-to-noise ratio. Unfortunately, these contrast agents are known for inducing changes in vascular anatomy like vascular distension or ruptures (Microfil) (Hong et al. 2020) and other artifacts such as discontinuous/incomplete filling of the capillaries and microvessels (Perrien et al. 2016) and inhomogeneous or weak signal, also due to particle aggregations (e.g., in the case with BaSo_4_ suspension) (Hlushchuk et al. 2018; Krucker et al. 2006; Perrien et al. 2016).

In this manuscript, we present a new postmortem angiography approach based on µAngiofil (Hlushchuk et al. 2018, 2019; Schaad et al. 2017), a novel polymer-based contrast agent. The latter has an excellent filling capacity as well as a radio-opacity, which is higher than the one of bone tissue, which allows visualization of the CNS vasculature even within the intact skull or the vertebral column. This approach gives the possibility of studying the angioarchitecture in different brain regions, spinal cord, choroid plexus, eye and the blood vessels of the dura mater or leptomeninges. In the present work, we show not only the different classical parts of the CNS, but also a promising organ for the research of the (neurodegenerative) CNS disorders that can be adequately visualized using microangioCT, i.e., the eye, which is considered “a window to the brain” (London et al. 2013).

The datasets obtained would plausibly empower a rather straightforward quantitative analysis of the structures, including the microvasculature. Moreover, the methodology introduced permits a correlative approach, i.e., microangioCT followed by classical histology, immunohistochemistry and even electron microscopy.

## Materials and methods

### Gliosarcoma rat model

All procedures related to animal care conformed to the Guidelines of the French Government with license 045998.03. Rats were induced anesthesia in a chamber containing 5% isoflurane in air prior to maintenance anesthesia with an intraperitoneal injection of xylazine/ketamine (64.5/5.4 mg.kg^−1^) mixture for tumor cell implantation.

### Other animals

Animal procedures were performed in accordance with the applicable Swiss legislation on the protection of animals and were approved by the corresponding cantonal veterinary offices. Two Göttingen minipigs, three Wistar rats, two Fischer rats and six C57BL/six wild-type mice were used in the study. Göttingen minipigs were obtained from Ellegaard (Dalmose, Denmark) and Wistar rats were obtained from Charles River (Sulzfeld, Germany). The minipigs and Wistar rats were housed at the animal facility of the Roche Innovation Center Basel (Basel, Switzerland) under appropriate conditions. Minipigs were anesthetized by a Zoletil mix for pigs [zoletil (1 bottle) + 6.25 ml xylazine (20 mg/ml) + 1.25 ml ketamine (100 mg/ml) + 2.5 ml butorphanol (10 mg/ml)] dosed at 1 ml per 15 kg. Wistar rats were anesthetized with a mixture of fentanyl (0.005 mg/kg), medetomidine (0.07 mg/kg) and midazolam (2 mg/kg). Both were afterwards euthanized with an overdose of intravenous pentobarbital and µAngiofil perfusion was performed postmortem. C57BL/6 mice were purchased from Janvier (Genest SaintIsle, France). Mice were housed in individually ventilated cages under specific pathogen-free conditions at the Theodor-Kocher Institute (Universität Bern, Bern, Switzerland).

### Perfusion with µAngiofil

The animals were perfused according to the procedure as described elsewhere (Hlushchuk et al. 2018; Schaad et al. 2017). Briefly, µAngiofil (Fumedica AG, Switzerland) was prepared according to the manufacturers’ instructions and left to stand for at least 20 min to get rid of the air bubbles. In the meantime, the animals were deeply anesthetized and heparinized. Subsequently, the artery suitable for the selective perfusion of the organ of interest was accessed and cannulated. The region of interest was then rigorously perfused with phosphate-buffered saline (PBS) to completely wash out the blood. Subsequently, the region of interest was perfused with µAngiofil with the help of the double-syringe injection pump (Fumedica AG, Switzerland) under visual control until the organ of interest turned blue (in case of CNS we could observe only the external tissues/organs: the eye, tongue, skin, etc.). By the end of the perfusion, the outflow was clamped, the mouse/rat/minipig was covered with a wet tissue and left for 20–30 min to let the µAngiofil polymerize. Afterward, the sample/organ of interest was gently excised and put into the fixative solution (2% paraformaldehyde solution in PBS) and stored in the cold room for post-fixation (at least over night). The samples were scanned at convenience at a later time point.

### Stereotaxic implantation of 9L tumor cells

Transplantable 9L cells were implanted in two 10-week-old Fischer rats weighing between 180 and 220 g. The implantation procedure adapted and followed the protocol described in Bouchet et al. (2014). Briefly, 4.10^4^ 9L cells suspended in 2.4 µl Dulbecco’s modified Eagle’s medium (DMEM) were injected using a Hamilton syringe into the right caudate nucleus (9 mm anterior to the ear bars, i.e., at the bregma, 3.5 mm lateral from the bregma, 5.5 mm depth from the skull surface) of anesthetized Fischer rats placed in a stereotactic head holder.

### MicroCT imaging and quantitative analysis

The harvested and post-fixed samples were afterward scanned using high-resolution microCT desktop scanners SKYSCAN 1172 or SKYSCAN 1272 (Bruker microCT, Kontich, Belgium). Before scanning, the rat and three of the mouse brains were removed from the skull. The spinal cord as well as some of the mouse brain samples stayed in situ surrounded by the bone (skull/vertebral column). The organs were scanned at 35 to 60 kV, 360 degrees rotation, frame averaging = ON, voxel size range 1.65–6.56 um. The resultant projections were used for the back-projection reconstruction using the NRecon Software (Bruker microCT). The 3D datasets obtained were then visualized using the CTvox software (Bruker microCT). For quantitative analysis of the vasculature, the subvolumes within the microangioCT datasets were selected (500 $\times$ 500 $\times$ 500 voxels): first—within the volume of interest (tumor), the other—on the contralateral side (non-tumor). The sub-datasets were then analyzed using CTan Software (Bruker microCT) to assess the vascular volume density and vessel diameter (“fitting spheres” approach). The segmented and analyzed subvolumes were subsequently visualized using CTvox software (Bruker microCT).

### Correlative imaging: semithin sectioning

After microCT scanning, the samples of interest (in our case, the rat eye) were further processed for correlative imaging as described elsewhere (Hlushchuk et al. 2011) . Briefly, the samples were refixed in 2.5% glutaraldehyde solution and then postfixed in 1% OsO_4_, dehydrated in ethanol array and embedded in epoxy resin. Subsequently, 0.8 µm-thick sections were obtained using glass knives, stained with toluidine blue and viewed under the light microscope (Zeiss Axio Imager M2). Resultant images were digitized using an Olympus UC50 camera.

## Results

### Brain vasculature: from overview to microvessel visualization

**Fig. 1:**
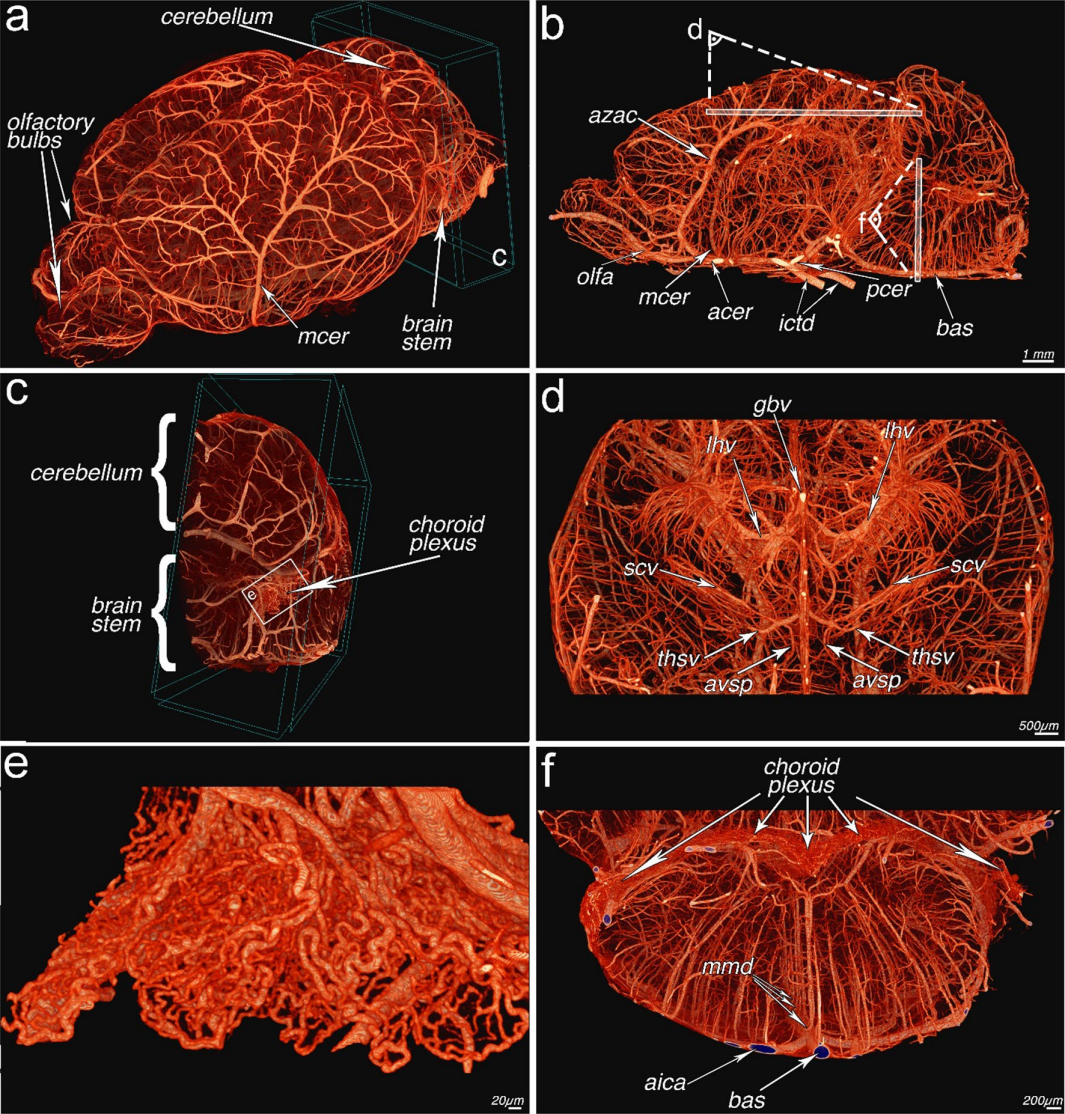
MicroangioCT of the murine brain. **a** Mouse brain vasculature overview including olfactory bulbs, brainstem and cerebellum. The artery shown on the lateral surface is the middle cerebral artery (mcer). **b** Virtual sagittal paramedian section through the brain vasculature. Arteries shown here are the basilar (bas), posterior cerebral (pcer), internal carotid (ictd), anterior cerebral (acer), middle cerebral (mcer), olfactory (olfa) and azygos anterior cerebral (azac). **c** Subvolume visualization as marked in ‘**a**’: partial representation of the vasculature of the cerebellum and brainstem with a notable outgrowth of the choroidal plexus of the fourth ventricle on the lateral surface. **d** Overview of the deep veins of the brain. The veins shown here are the thalamostriate (thsv), superior choroidal (scv), longitudinal hippocampal (lhv), great brain (gbv) and anterior vein of septum pellucidum (avsp). **e** Illustration of the choroid plexus from “**c**” at higher resolution. **f** Partial view of the vasculature of the brainstem as indicated in “**b**”. Arteries shown here are the basalis (bas), median medullary (mmd) and anterior inferior cerebellar (aica)

The approach suggested is well suited for the visualization of the brain of laboratory rodents. µAngiofil polymerizes in the blood vessels after injection and, once polymerized, serves as a robust scaffold and the animal brains can easily be removed from the skull. Removal of the skull reduces the size of the scanned sample and its density. It leads to a potentially higher-resolution scan with less noise/shorter scanning time. Moreover, it is an unavoidable step if one wants to scan single parts/regions within the brain at the highest possible resolution, which is not yet possible within the intact skull. After the removal from the skull and due to the high radio-opacity of the µAngiofil, the vessels of the mouse brain could be visualized without any remarkable image post-processing (Fig. 1). Not only vessels adjacent to the surface (Fig. 1a), but also the ones deep inside the brain could be unambiguously imaged. Figure 1 displays the overview of the whole mouse brain and vascular details of different sub-regions, including the choroid plexus and microvasculature of the brainstem. The methodology reported here clearly allows 3D visualization of the brain vasculature and the possibility to “zoom in” to the capillaries (diameter 4–10 µm) within the region of interest.

### Brain and spinal cord within the intact bony shell

**Fig. 2:**
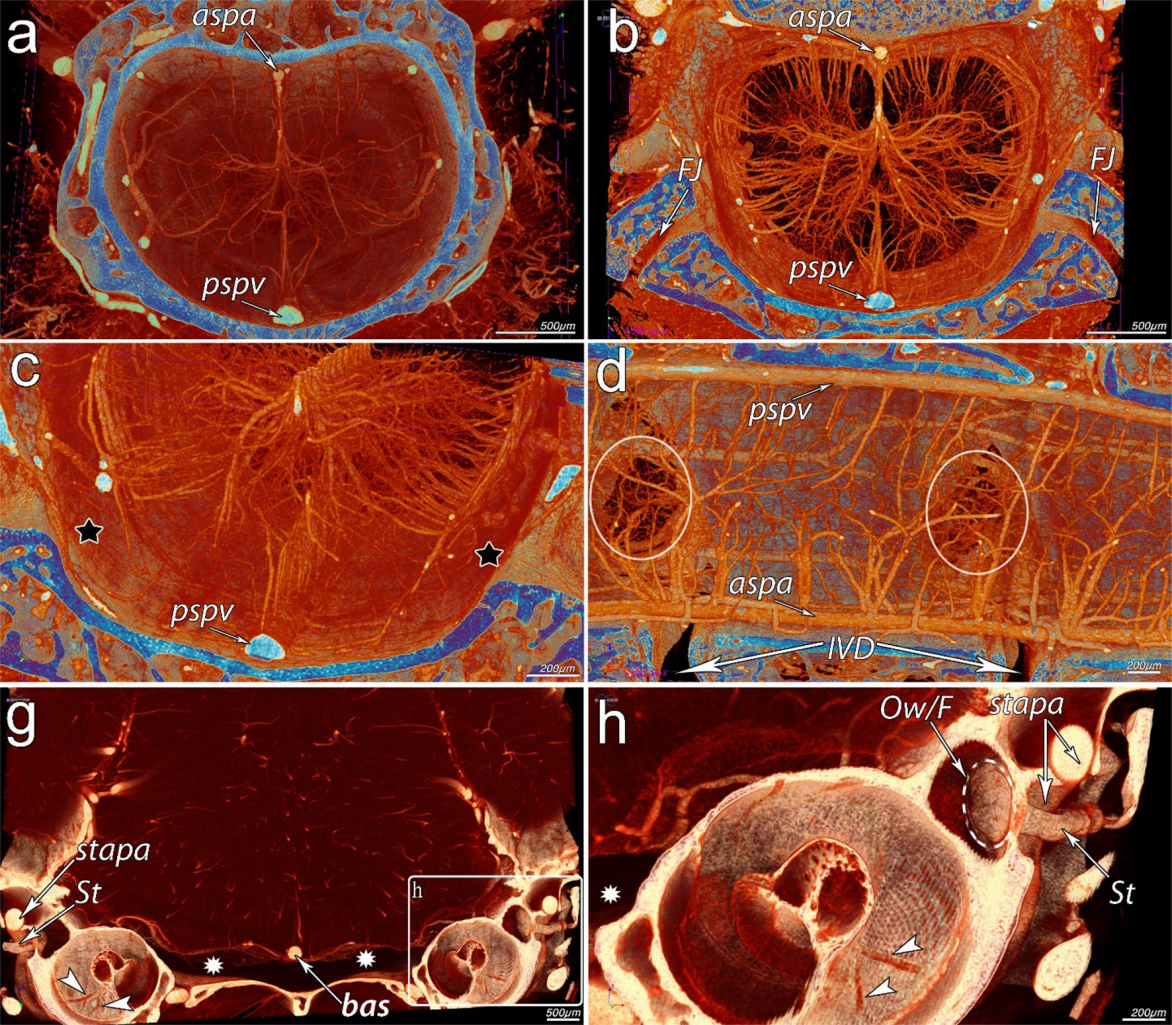
MicroangioCT of the CNS vasculature within the intact bone shells. Lumbar segment (L1-L2) of the murine spinal cord (**a**–**d**). Virtual section through brainstem and inner/middle ear (**g**-–**h**). **a** Overview from the cranial aspect of the spinal cord vasculature and the vertebral column; anterior spinal artery (aspa) is on the top and posterior spinal vein (pspv) at the bottom of the image. **b** View from the caudal aspect of the spinal cord with the “butterfly” pattern of the microangioarchitecture, indicating the highest density of the capillary network within the gray matter of the spinal cord. FJ indicates the intervertebral facet joints. **c** A closer look into the epidural space (marked with black asterisks) between the bony channel and the spinal cord with bigger veins lying at the inner surface of the vertebrae. **d** Lateral view of the blood supply to the spinal cord; IVD indicating avascular intervertebral discs. The white circles denote intervertebral foramina. **g** Bird’s eye view of the cochleae and brainstem with basal artery (bas) situated at the median line within the subarachnoid space (asterisks in **g**). Lateral to both cochleae, one can clearly see the stapes (St) with stapedial artery (stapa) going through its foramen, between the posterior and the anterior crus of the stapes. **h** The oval window/footplate of the stapes (Ow/F) as well as small radiating arterioles (white arrowheads) supplying the cochlea is clearly visible

The contrast-enhanced microCT by µAngiofil had apparently no substantial difficulties to visualize the microvasculature of the spinal cord as well as brainstem within the bony vertebral column (Fig. 2). One could recognize the “butterfly” pattern of the spinal cord corresponding to the gray matter (= increased capillary density). This approach enables the visualization of the microvessels not only deep within the spinal cord tissue, but also with the intact bony shell as well as inside the bony structures (see Fig. 2g, h). The presence of bony compartments allows, for the first time, in situ evaluation of the vasculature and its relationship to the surrounding skeletal structures (see Supplemental Video 1).

### Quantification of neoangiogenesis in a rat model of gliosarcoma

**Fig. 3:**
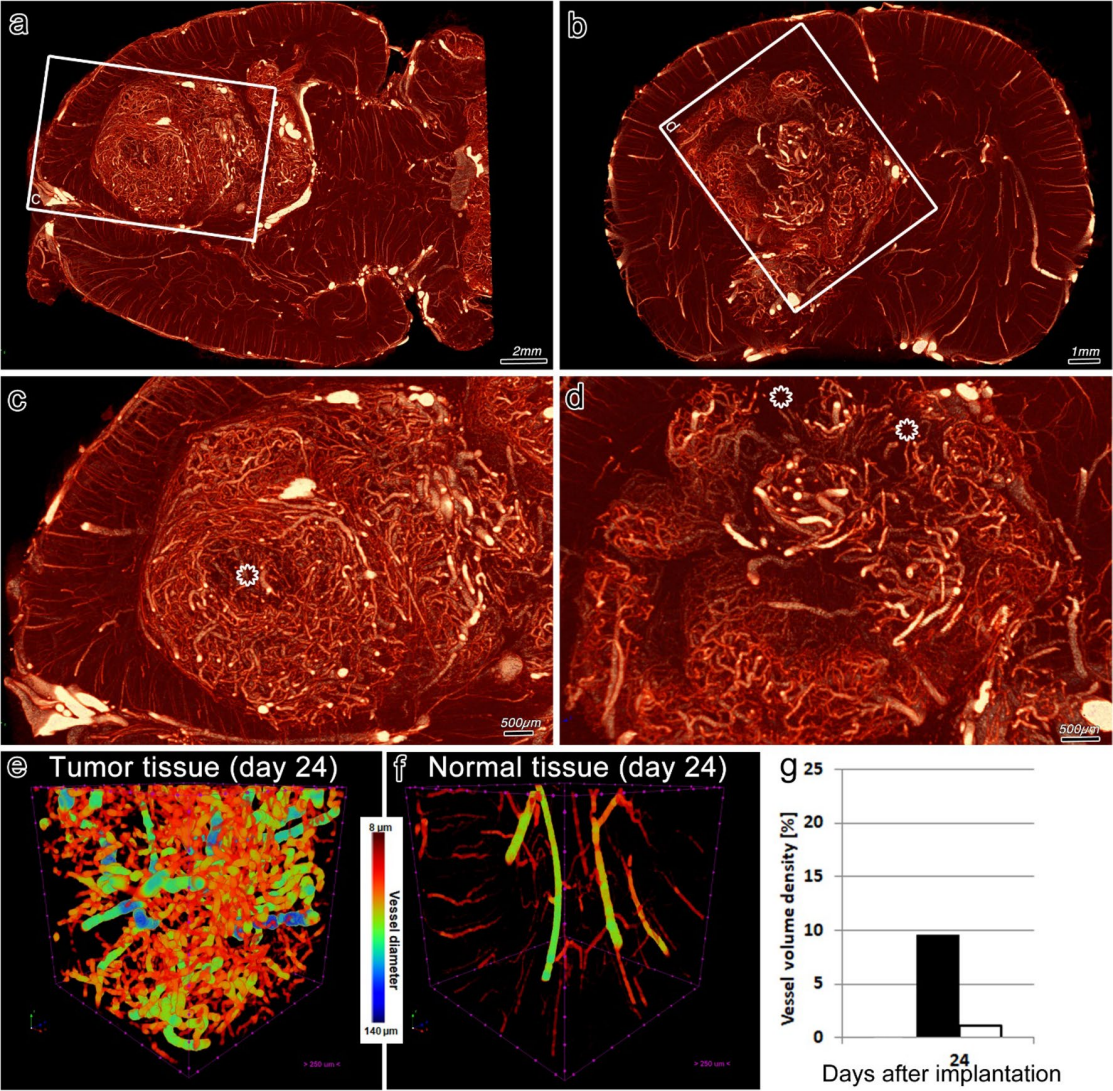
MicroangioCT of 9L gliosarcoma in a rat brain. Upper panels display virtual horizontal (**a**) and transverse (**b**) sections through the vasculature of a rat brain containing a 9L gliosarcoma at low magnification. **c**, **d** Regions framed in the upper panels at higher magnification. The region affected by a tumor can be easily detected by its characteristic vascularization pattern. Note the inhomogeneities of the vascular pattern within the gliosarcoma—the regions with lower vascular density, or even avascular (asterisks) alternate with high density regions. Quantitative analysis is presented in panels. **e–g** The analyzed vasculature of the subvolumes (500 × 500 × 500 voxels) within the tumor (**e**) and normal brain tissue (**f**) are presented with color coding according to the vessel diameter (see the scale in the figure), clearly demonstrating that the tumor vessels are irregular, dilated and in higher numbers in comparison to normal tissue. **g** Quantification of the vessel volume density: black bar indicates vessel volume density within the tumor, white bar—in normal brain tissue

The 9L gliosarcoma rat brain model was used to verify the suitability of µAngiofil-based microangioCT approach for visualization and quantification of the tumor vasculature. The sample represented in Fig. 3 shows the vasculature of a rat brain with 9L gliosarcoma at 24 days after tumor cells implantation (no treatment), including vascular inhomogenities within the tumor volume (regions marked with asterisks in Fig. 3c, d).

The imaging performed here provides insights into the location and size of the tumor through its characteristic vascular pattern with dilated vessels and much higher vessel density compared to the surrounding tissue. The high radio-opacity of the µAngiofil-filled vessels enables a straightforward segmentation of the vessels to distinguish them from the soft tissues. The segmented datasets can then be easily visualized and quantified without additional complicated image post-processing (Fig. 3e–g).

### Correlative approach combining microangioCT with histology

**Fig. 4:**
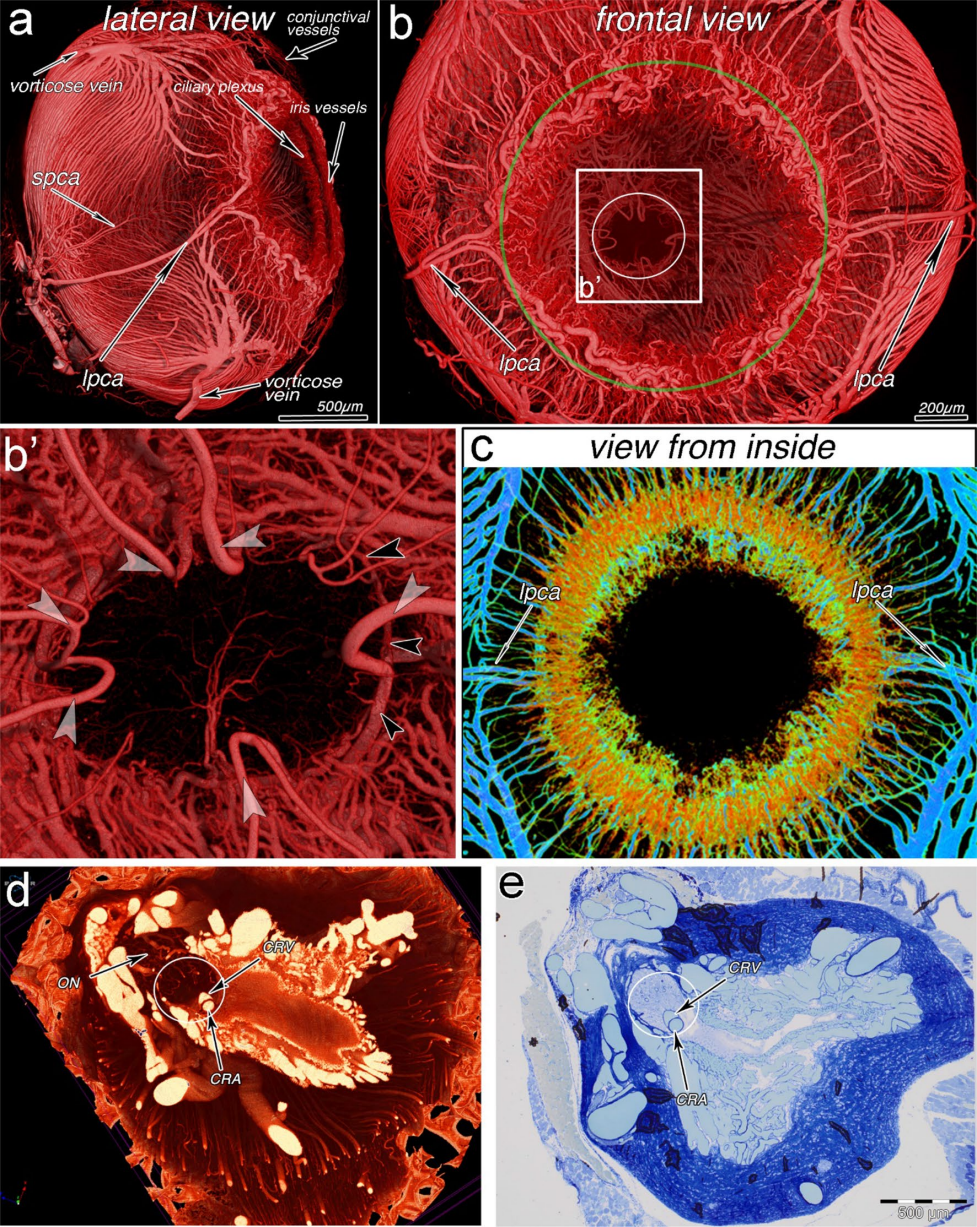
MicroangioCT of the minipig eye (**a**–**c**) and correlative imaging of the optical disc and bottom of the rat eye (**d**, **e**). **a**, **b **External views of the vasculature of the minipig eyeball. The vessels indicated are conjunctival arteries and veins, vorticose veins, short and long posterior ciliary arteries (spca and lpca) as well as iris vessels and the ciliary plexus. **b** The green circle indicates the major arterial circle of the iris; the vessels within the green circle, including **b’**, are from the dorsal part of the eye. White circle marks the optic nerve head region and entrance of retinal vessels into the eyeball; **b’** shows the region framed in **b** at higher magnification: a partial arterial circle of the optic nerve (analogy to circle of Zinn–Haller found in humans) is indicated with black arrowheads. **c** Dorsal view through the iris, i.e., from the position inside the eyeball. The CTvox transfer function in panel **c** is different from the one in panels **a** and **b** providing the idea about the size of presented vessels (bigger and medium-sized vessels: blue. Small ones: yellow-orange). **d**, **e** Correlative imaging of the Wistar rat eye: **d**—the virtual section through the microangioCT-dataset (isotropic voxel side size 0.65 µm) near the entrance point of the optic nerve (circle in **d** and **e**). One can clearly recognize the central retinal artery (CRA) and vein (CRV) as well as small vessels feeding the optic nerve (ON). (**e**) Light microscopy image of a semithin section corresponding to the area represented in “**d**”

Tracking of the vascular hierarchy is essential for evaluation of the organ angioarchitecture. The lateral and frontal view of the minipig eye represent ocular vessels such as vorticose vein, short and long ciliary arteries, ciliary plexus, and circulus arteriosus iridis major and minor (Fig. 4a–c). The latter vessel supplies the iris micovasculature, which is characterized by alternating vessel size. The aforementioned possibility to do a straightforward 3D-analysis/visualization of the vessels is crucial for understanding vascular hierarchy and vascular alterations along the supplying and draining organ vessels (Fig. 4c). Additional crucial issue for preclinical as well as postmortem clinical imaging techniques is the possibility for validation and correlation by another technique such as histological investigation. To test this possibility, a microangioCT dataset of an eye, which is a challenging organ for correlative imaging (Sengle et al. 2013), was obtained. After the tomographic imaging, a classical histological investigation was performed (Fig. 4d,e), which allowed to compare the microangioCT findings with the histological ones. Such correlative approach allows follow-up also by electron microscopy (data not shown).

## Discussion

The traditional optical imaging techniques like two-photon laser scanning microscopy or ultrastructural imaging such as scanning or transmission electron microscopy are able to visualize tissues at the sub-micrometer scale. However, they are incapable of providing visualization of the whole cerebral vasculature at a 3D level. Laser scanning microscopy is limited by its low penetration depth and sample size. Vascular corrosion casts combined with scanning electron microscopy methods are destructive and can only provide a view of the surface of the sample (Hlushchuk et al. 2019).

Nonetheless, in the last few decades, great progress has been made in many techniques used to image whole-CNS vascular structures and a few novel approaches have emerged.

Light-sheet microscopy (LSM), in combination with constantly improving tissue clearing methods, has proven to be a useful tool in neuroscience to image whole mouse brain (Mullenbroich et al. 2018; Pan et al. 2016). However, residual opaque objects which are always present to some extent even in extremely well optically cleared samples cause stripe artifacts, which severely affect image homogeneity in the best case, and, in the worst case, completely obscure features of interest (Mullenbroich et al. 2018). The major drawback of the approach is that since the skull and bones need to be removed for LSM imaging, it is not possible to visualize vessels within leptomeningeal layers, dura mater or inner ear. Another remarkable limitation of the approach is its anisotropic resolution: although its lateral resolution approximates 1 µm, the axial (*Z*) resolution is much worse (Mullenbroich et al. 2018). Further drawback is that ultrastructural examinations, such as with electron microscopy, are not possible after LSM imaging.

The micro-optical sectioning tomography (MOST) is a relatively new promising technology, but, regrettably, also of a destructive nature. Brains have to be removed from the skull and processed further: the embedded whole brain specimens are imaged by performing simultaneous thin sectioning and imaging. For each mouse brain, uninterrupted sectioning and imaging may take up to 7 days and produce up to 2.4 terabytes of data (Xiong et al. 2017). While imaging with MOST is very time-consuming (10 days for one mouse brain), the voxel size possible is remarkably better at 0.3 $\times$ 0.3 $\times$ 1 µm, albeit still anisotropic.

Another recently established approach is the synchrotron radiation phase-contrast tomography (SR-PCT) which can retrieve the information in the samples based on phase-retrieval algorithms (Burvall et al. 2011). So far, the resolution that was achieved while scanning whole brain or spinal cord samples with SR-PCT enables resolving the microvessels of around 10 µm in diameter [voxel size 5.92 μm in brain (Zhang et al. 2015)] or 3.7 µm in the spinal cord (Hu et al. 2015). This method will most probably get better with time considering the expected improvements at the sensor side. It is a non-destructive method that does not require a contrast agent, although the resolution is limited to the microvessels of at least 10 µm in diameter. For smaller vessels, the use of a contrast agent is at the moment unavoidable. Limited access to synchrotron sources is the major drawbacks of the SR-PCT. Additionally, it is possible to image biological samples with grating-based imaging to access the phase information inside the samples. This hardware-based phase contrast approach is limited in resolution though. Generally, X-ray phase imaging with a grating interferometer allows for spatial resolutions down to a few microns (Weitkamp et al. 2005)*.* To our best knowledge, there is currently only one system commercially available (Bruker SkyScan 1294), while other systems for grating-based phase-contrast imaging rely on preclinical and prototype systems (Bech et al. 2013; Stutman et al. 2011; Tapfer et al. 2012; Yaroshenko et al. 2013).

The microangioCT approach in this study provides the possibility to evaluate the entire vasculature of the central and peripheral nervous system down to the capillaries by imaging the samples using a desktop microCT machine (Figs. 1, 2). The resolution achieved is comparable to the one published for the synchrotron studies (Cao et al. 2017).

Moreover, due to the radio-opacity of µAngiofil, the perfused vasculature within the bone tissue can be visualized (Fig. 2 as well as Supplemental Video 1). Until now, the visualization of the vasculature within the bone has been a major challenge, which can only be partially overcome by sophisticated protocols with severe drawbacks, including sample destruction (Schneider et al. 2009). The suggested approach with µAngiofil enables investigation of the pathophysiological vascular changes and malformations, which may take place within the bony shells. For example, abnormal cochlear microcirculation is considered a potential cause in noise-induced hearing loss, age-related hearing loss (presbycusis), sudden hearing loss or vestibular function, and Meniere’s disease (Shi 2011).

The option of 3D imaging of the CNS blood vessels in the intact skull and the vertebral column is of special relevance when considering the different properties of those blood vessels. While the blood vessels of the dura mater do not establish a blood–brain barrier (BBB), those in the subarachnoid space establish barrier characteristics although they are not associated with astrocytic end-feet as described for CNS microvessels establishing the BBB within the CNS parenchyma (Liebner et al. 2018). The possibility of exploring the precise angioarchitecture of the leptomeninges and the CNS parenchyma in the context of the intact bony compartments is of further interest, when considering immune cell trafficking to the CNS during immune surveillance or in neuroinflammation as the leptomeningeal barriers separate compartments in the CNS that differ with respect to their accessibility of immune cells (Engelhardt et al. 2017).

The option to leave the brain in the skull enables a straightforward immobilization of the sample without squeezing/deforming it and the surface vessels remaining intact. Furthermore, it minimizes sample dehydration/shrinkage and corresponding imaging artifacts during the long-lasting high-resolution microCT scans. On the contrary, outside the brain cage, the brains are very susceptible to dehydration, which results in sample shrinkage and imaging artifacts, which are evident especially in the higher magnifications as a feathering (or shadowing) of the images (Hong et al. 2020). To mitigate this problem, the researchers often use wet paper tissue or wrap the samples with cling film. This reduces the dehydration, but wrapping and unwrapping of the exposed brain often damage the surface vessels (Hong et al. 2020).

Besides superior imaging, the radio-opacity of µAngiofil allows reliable evaluation and quantification of the vasculature in a short time, without further complicated image post-processing (Fig. 3). Since this approach is non-destructive, it enables researchers to ping pong the vascular phenotype with protein expression (e.g., IHC) or local morphology (Fig. 4).

The microangioCT approach with µAngiofil renders the common desktop microCT scanners a promising everyday tool for the evaluation of the (micro)vasculature of the central nervous system in preclinical and basic research.

## Electronic supplementary material

Below is the link to the electronic supplementary material.

Supplementary file1 (MP4 386827 kb)
